# Architecture of the Triceps Surae Muscles Complex in Patients with Spastic Hemiplegia: Implication for the Limited Utility of the Silfverskiöld Test

**DOI:** 10.3390/jcm8122096

**Published:** 2019-12-01

**Authors:** Kun-Bo Park, Sun Young Joo, Hoon Park, Isaac Rhee, Jong-Kwan Shin, Sharkawy Wagih Abdel-Baki, Hyun Woo Kim

**Affiliations:** 1 Division of Pediatric Orthopaedic Surgery, Severance Children’s Hospital, Yonsei University College of Medicine, Seoul 03722, Korea; pedoskbp@yuhs.ac; 2 Department of Orthopaedic Surgery, Incheon St. Mary’s Hospital, The Catholic University of Korea College of Medicine, Incheon 21431, Korea; jsy9528@gmail.com; 3 Department of Orthopaedic Surgery, Gangnam Severance Hospital, Yonsei University College of Medicine, Seoul 06273, Korea; hoondeng@yuhs.ac (H.P.); jkshin2403@yuhs.ac (J.-K.S.); 4 Medical course, University of Melbourne Melbourne Medical School, 3010 Melbourne, Australia; isaac.rhee@gmail.com; 5 Department of Orthopaedic Surgery, Aswan University Hospital, Aswan University Faculty of Medicine, Aswan 81528, Egypt; sharkawyelbanna@aswu.edu.eg

**Keywords:** architectural properties, triceps surae, cerebral palsy, limited utility, the Silfverskiöld test

## Abstract

The Silfverskiöld test has long been used as an important tool for determining the affected muscles of the triceps surae in patients with equinus deformity. However, the test may not reflect the altered interactions between the muscles of the triceps which are affected by spasticity. The purpose of this study was to compare the architectural properties of the triceps surae muscles complex using ultrasonography, between hemiplegic patients and typically-developing children. Specifically, we wished to examine any differences in the architecture of the three muscles with various angle configurations of the knee and ankle joints. Ultrasound images of the medial gastrocnemius, lateral gastrocnemius, and soleus were acquired from paretic (group I) and non-paretic (group II) legs of ten patients and the legs (group III) of 10 age-matched normal children. A mixed model was used to evaluate the differences in the measurements of muscle architecture among the groups and the effects of various joint configurations on the measurements within the muscles. Compared to the results of measurements in groups II and III, the fascicle length was not different in the medial gastrocnemius of a paretic leg but it was longer in the lateral gastrocnemius and shorter in the soleus; the pennation angle was smaller in both medial and lateral gastrocnemii and was not different in the soleus; and the muscle thickness was found to be reduced in the three muscles of the paretic leg. Contrary to the observations in both the medial and lateral gastrocnemii, the fascicle length was increased and the pennation angle was decreased in the soleus with an increase of knee flexion. Through the current simulation study of the Silfverskiöld test using ultrasonography, we found that the changes detected in the architectural properties of the three muscles induced by systematic variations of the position at the ankle and the knee joints were variable. We believe that the limited utility of the Silfverskiöld test should be considered in determining an appropriate operative procedure to correct the equinus deformity in patients with altered architecture of the muscles in conditions such as cerebral palsy, as the differing muscle architectures of the triceps surae complex may affect the behavior of the muscles during the Silfverskiöld test.

## 1. Introduction

An equinus gait is the most common abnormality seen in patients with cerebral palsy (CP). Although conservative treatments are typically used first, most patients with an equinus deformity eventually require surgical correction, in which the calf muscles are lengthened based on the assumption that the ankle plantarflexors are abnormally shortened. The triceps surae muscles are a structurally complex unit, consisting of the soleus (a one-joint muscle crossing the ankle joint) and the medial and lateral gastrocnemius muscles (two-joint muscles crossing both the knee and ankle joints). 

The Silfverskiöld test (Figure S1) is commonly used as an important clinical tool for determining the affected muscles of the triceps in patients with equinus deformity [1,2]. When ankle dorsiflexion is facilitated by the bending of the knee joint (Silfverskiöld test-positive), surgery aiming to only lengthen the gastrocnemius muscle is required; otherwise (Silfverskiöld test-negative), surgeons typically lengthen the Achilles tendon with the assumption that both the gastrocnemius and soleus have been shortened. However, the Silfverskiöld test may not reflect the altered interactions between the muscles of the triceps which are affected by spasticity. For example, a Silfverskiöld test-positive patient may demonstrate simultaneous activation of both the soleus and gastrocnemii, as observed in an electromyographic study [3]. In addition, CP patients with an equinus gait may have longer-than-normal Achilles tendons and shorter-than-normal muscle bellies [4,5]. As such, lengthening of the tendo-achilles based on the suggestions by Silfverskiöld would change the ratio of muscle and tendon length and affect the competency of the plantar flexion–knee extension couple. Furthermore, variable knee flexion angles may influence the excursion of the soleus through the action of the aponeurosis of gastrocnemius [6,7], resulting in difficulties in interpreting the findings of the test.

Lengthening of the triceps surae muscles to correct equinus deformity is the most commonly performed operative procedure in CP. However, the literature remains confusing regarding even the definition of isolated gastrocnemius or combined gastrocnemius soleus tightness, with the maximal degrees of ankle dorsiflexion used as a measure of plantar flexion deformity ranging from 0° to 25° [8]. Furthermore, to date, there is no universal standard or consensus as to the appropriate degrees of knee flexion needed during the test and the minimal degrees of ankle dorsiflexion detected at each corresponding angle of knee flexion to determine the affected muscles responsible for the equinus; we believe this lack of standardized information since the introduction of the Silfverskiöld test is due to the possibility of contribution of both the gastrocnemius and soleus to differing degrees. Practically, it may be difficult to conceptualize a simple clinical situation in which the gastrocnemius is tight but the soleus is lax and vice versa, especially in patients with CP. Contrary to an ankle contracture caused by non-neurologic conditions, adequate treatment for the correction of an equinus gait in CP may require a more comprehensive understanding of the spasticity-induced structural-functional alterations of the triceps [9,10]. 

The architectures of the skeletal muscles have been studied in vivo using ultrasonography [11,12,13,14,15,16], and recent studies suggested that the treatment protocol for the muscle deformity in CP should be individualized with the considerations of alterations in the muscle architecture [10,17,18,19,20,21,22]. Previous studies have investigated the differences in muscle architectures between typically-developing children and children with CP. However, they studied the architectural properties of the triceps surae with the knee and ankle at the resting position [19,22,23,24,25,26,27,28]. Furthermore, their findings are not comparable due to disparities among the studies, in terms of the presence of an accompanying skeletal deformity, inclusion of the patients with a wide spectrum of neurologic involvement and varying ambulatory statuses. 

The purpose of our study was to compare the architectural properties of the triceps surae muscles complex using ultrasonography, between the patients with spastic hemiplegic cerebral palsy (SHCP) and age-matched typically-developing children. Specifically, we wished to examine any differences in the architecture of the three muscles with various angle configurations of the knee and ankle joints. Through this approach we reappraised the Silfverskiöld test in patients with equinus deformity. 

## 2. Experimental Section

### 2.1. Subjects

This study was approved by the institutional review board of the Severance Hospital (Seoul, Korea). The pediatric patients with equinus gait and SHCP and the age-matched normal children with typical development were recruited through the outpatient clinic. For SHCP patients, the inclusion criteria were patients with an ability to walk defined by the Gross Motor Function Classification System (GMFCS) level I [29]; type II hemiplegia by the classification system of Winters et al., characterized by a persistent ankle plantarflexion during the stance and swing phases [30]; and no history of previous treatment for ankle equinus (e.g., selective posterior rhizotomy, orthopaedic surgery, or botulinum toxin injection). The exclusion criteria included patients with static or dynamic hamstrings tightness, and a fixed ankle plantarflexion contracture. Additionally, as the functional competence of the ankle plantarflexors may be affected by the associated lever-arm diseases which may develop in the coronal and transverse planes as well, we excluded the patients with a concomitant skeletal abnormality of the lower extremity such as equinovarus or planovalgus foot deformity, and rotational malalignment as determined by the trochanteric prominence angle test for femoral anteversion and the thigh-foot angle for tibial torsion.

Ten children with SHCP (6 boys and 4 girls; average age, 7 years 1 month; age range, 5–10 years 1 month) and another 10 age-matched children with typical development (6 boys and 4 girls; average age, 6 years 7 months; age range, 5 years 2 months–8 years) were enrolled in the study. In total, the study sample consisted of 10 paretic legs (group I), 10 non-paretic contralateral legs (group II), and 20 normal legs of typically-developing children (group III).

### 2.2. Procedure

During the ultrasonographic evaluation, the subjects lay prone on the examination table and were instructed to refrain from moving the ankle and knee joints. For the simulation of the Silfverskiöld test, we used a total of 12 joint configurations, consisting of a combination of three knee joint angles (0°, 45°, and 90° of flexion) and four ankle joint angles (10° of dorsiflexion; 0°, 15°, and 30° of plantarflexion). Each joint configuration was created using an electric goniometer (Biometrics Ltd, Cwmfelinfach, Gwent, UK) and fixed with an adjustable half-cast supporting device surrounding the anterior aspect of the leg.

Conventional ultrasound examinations were performed using a linear phased array transducer with a frequency of 5–13 MHz (HI VISION Avius; Hitachi Medical, Chiba, Japan), and longitudinal ultrasound images of the medial gastrocnemius (MG), lateral gastrocnemius (LG), and soleus (SOL) were acquired from the muscle midbelly. The methods of probe positioning and the measurement of muscle architectures were standardized as described by others [11,14,21,24,31,32]. Briefly, a transducer was first placed at around one thirds (for MG and LG) or half (for SOL) of the distance between the popliteal crease and the center of the lateral malleolus, and then its placement was adjusted so as to detect the maximum anatomic cross-sectional area of each muscle. The ultrasound probe was then rotated and/or tilted, and in order to minimize probe orientation-related errors we aligned the probe perpendicular to the deep aponeurosis within the muscle belly and used that alignment for subsequent longitudinal imaging of the path of muscle fascicles. After the examination of the gastrocnemius, we examined the position of the greatest thickness around the lateral half of the posterior soleus [11]; this was the proximal lateral fibers according to the description by Martin et al. [11,31]. To ensure adequate evaluation of muscle fascicle, the plane of the ultrasonogram was chosen parallel to the fascicles, which was verified by visualizing the fascicles along their lengths from the superficial to deep aponeurosis of the gastrocnemius and from the posterior aponeurosis to anterior aponeurosis of the soleus. 

**Figure 1:**
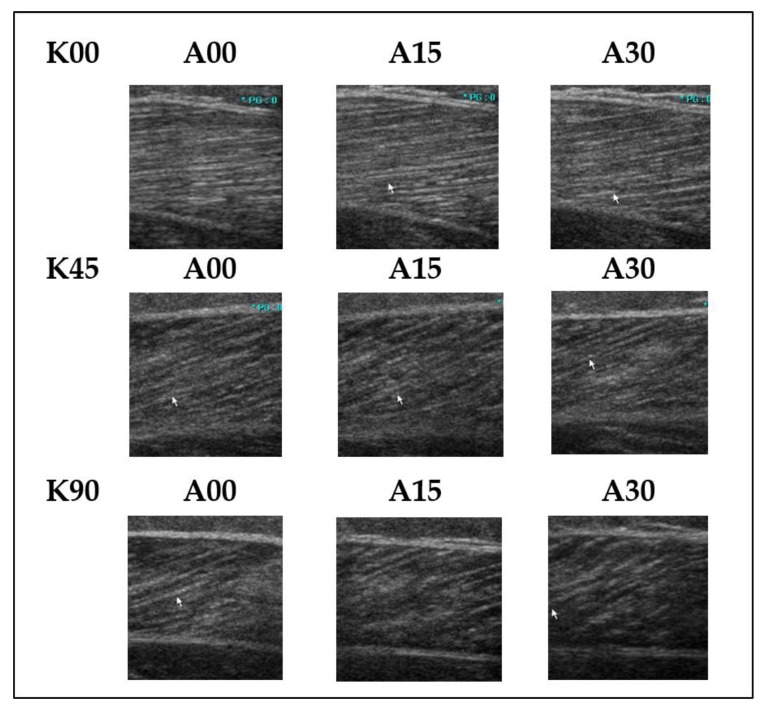
Ultrasonographic images of a lateral gastrocnemius (LG) of a paretic leg. A00, A15, and A30 represent ankle neutral, 15° of plantarflexion, and 30° of plantarflexion, respectively; while K00, K45, and K90 represent full knee extension, 45° of knee flexion, and 90° of knee flexion, respectively.

In group I, ultrasound images were not acquired at 10° of dorsiflexion due to the spasticity of the ankle plantarflexors. In total, 27 ultrasonographic images from the paretic legs and 36 images from the non-paretic contralateral legs and normal legs of the control group were captured and used for analysis (Figure 1).

### 2.3. Variables Describing Muscle Architecture

**Figure 2:**
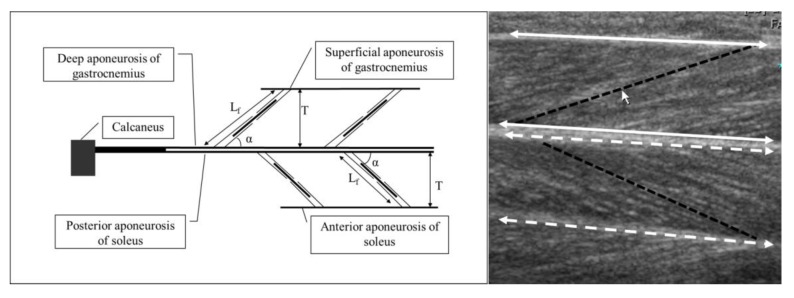
Schematic and real ultrasound images of the muscle architecture: fascicle length (Lf); pennation angle (Pa); and muscle thickness (T). On the right image: white solid lines indicate aponeuroses of the lateral gastrocnemius and white dashed lines indicate aponeuroses of the posterior soleus; and black dashed lines indicate fascicle length.

With use of the acquired ultrasound images, fascicle length (Lf), pennation angle (Pa), and muscle thickness (T) of the MG, LG, and SOL were measured by image-processing software (Image J, version 1.37; National Institutes of Health, Bethesda, MD, USA). Lf was defined as the distance between the muscle origin (where the fascicle and superficial aponeurosis of the gastrocnemius or posterior aponeurosis of the soleus meet) and insertion (where the fascicle and deep aponeurosis of the gastrocnemius or anterior aponeurosis of the soleus meet). Pa represented the angle formed between the fascicular path and the aponeuroses of the muscles; T, the shortest distance between the aponeuroses (Figure 2).

### 2.4. Statistical Analysis

All images were analyzed two times, and each variable describing the muscle architecture was measured by one of us who were blinded to the joint configurations. Statistical analyses were performed using SAS version 9.1 (SAS Institute, Cary, NC, USA). Mann–Whitney *U*-test and Fisher’s exact test was used to compare demographic data between the groups. A mixed model was used to allow repeated measurements in the individuals, and to evaluate the differences in the measurements of muscle architecture among the groups and the effects of various joint configurations on the measurements within the muscles, respectively. In addition, to adjust for the effects of age, sex, and height, we performed multivariate analysis. The level of significance was set at *p* < 0.05.

## 3. Results

### 3.1. Study Sample

There were no statistical differences in sex (*p* = 0.648), age (*p* = 0.143), height (*p* = 0.247), weight (*p* = 0.280), femoral anteversion (*p* = 0.841), or tibial torsion (*p* = 0.183) (Table 1). Details of Lf (mm), Pa (degree) and T (mm) measured at each joint angle configuration are presented in Table S1. Lf, Pa and T of individual cases are presented in Figures S2–S4.

### 3.2. Comparison of Fascicle Length (Lf)

**Figure 3:**
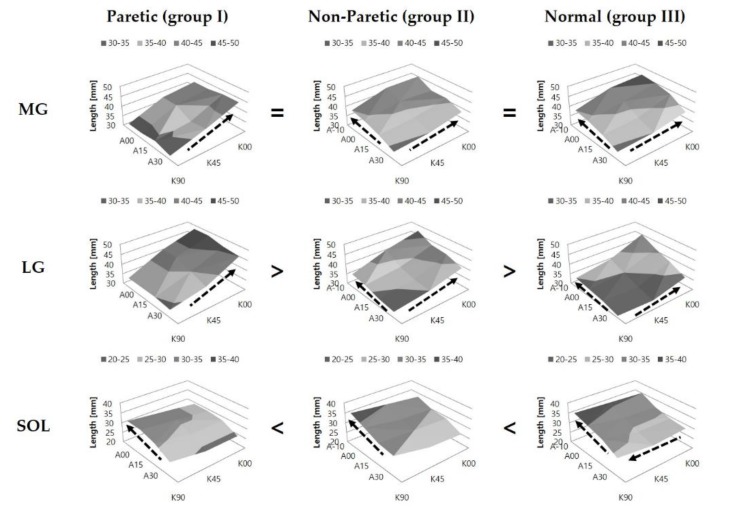
Fascicle length of the medial gastrocnemius (MG), lateral gastrocnemius (LG), and soleus (SOL) muscles at various configurations of the ankle and knee joint angles. The arrow indicates a significant increase in fascicle length. The symbols (=, >, <) indicate significant differences in fascicle length among the groups. A-10, A00, A15, and A30 represent 10° ankle dorsiflexion, ankle neutral, 15° plantarflexion, and 30° plantarflexion, respectively; while K00, K45, and K90 represent full knee extension, 45° knee flexion, and 90° knee flexion, respectively.

Fascicle length (Lf) of LG in group I (*p* < 0.001) and that of SOL in group III (*p* < 0.001) were longer than their respective values of the other groups. No significant difference in Lf of MG was noted among the groups (*p* = 0.564). In MG and LG, Lf was found to be increased with the increase of knee extension in all groups (group II, *p* = 0.002 in MG; others, *p* < 0.001). However, in SOL, the effect of knee position was observed to be significant in group III (*p* = 0.004) only, showing an increase of fascicle length with knee flexion. Lf was observed to be increased with the increase of ankle dorsiflexion in the three muscles of the triceps surae (group I, *p* = 0.001 in SOL; others, *p* < 0.001), except in MG (*p* = 0.799) and LG (*p* = 0.432) in group I (Figure 3). 

### 3.3. Comparison of Pennation Angle (Pa)

**Figure 4:**
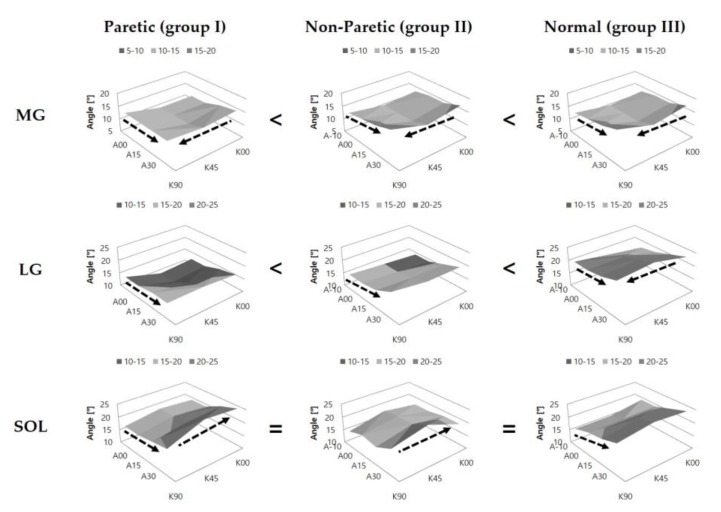
Pennation angles of the medial gastrocnemius (MG), lateral gastrocnemius (LG), and soleus (SOL) muscles at various configurations of the ankle and knee joint angles. The arrow indicates a significant increase in pennation angle. The symbols (=, >, <) indicate significant differences in pennation angle among the groups. A-10, A00, A15, and A30 represent 10° ankle dorsiflexion, ankle neutral, 15° plantarflexion, and 30° plantarflexion, respectively; while K00, K45, and K90 represent full knee extension, 45° knee flexion, and 90° knee flexion, respectively.

Pennation angle (Pa) of MG (*p* < 0.001) and LG (*p* < 0.001) in group III were the greatest. In SOL, no significant differences were noted among the groups (*p* = 0.396). In MG, Pa was observed to be decreased with the increase of knee extension in all groups (group I, *p* = 0.003; groups II and III, *p* < 0.001). In LG, Pa decreased with the increase of knee extension in group III only (*p* < 0.001). However, Pa of SOL increased with knee extension in groups I (*p* = 0.024) and II (*p* = 0.035). Pa decreased with the increase of ankle dorsiflexion in MG (groups I, II, and III, *p* = 0.038, < 0.001, and < 0.001, respectively), LG (groups I, II, and III, *p* = 0.002, < 0.001, and < 0.001, respectively), and SOL (groups I and III, *p* < 0.001 and < 0.001, respectively; group II, *p* = 0.071) (Figure 4).

### 3.4. Comparison of Muscle Thickness (T)

**Figure 5:**
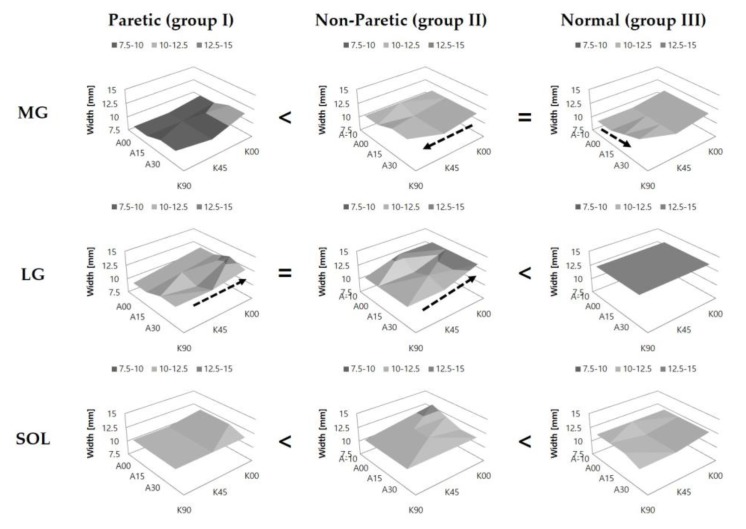
Muscle thickness of the medial gastrocnemius (MG), lateral gastrocnemius (LG), and soleus (SOL) muscles at various configurations of the ankle and knee joint angles. The arrow indicates a significant increase in muscle thickness. The symbols (=, >, <) indicate significant differences in muscle thickness among the groups. A-10, A00, A15, and A30 represent 10° ankle dorsiflexion, ankle neutral, 15° plantarflexion, and 30° plantarflexion, respectively; while K00, K45, and K90 represent full knee extension, 45° knee flexion, and 90° knee flexion, respectively.

Muscle thickness (T) of MG (*p* < 0.001), LG (*p* < 0.001), and SOL (*p* < 0.001) was the smallest in group I. T of MG was observed to be increased with the increase of knee flexion in group II (*p* = 0.001). T of LG increased with the increase of knee extension in groups I (*p* = 0.008) and II (*p* = 0.001), respectively. In SOL, the effect of knee position was not significant in all groups. The effect of ankle position was observed to be significant in MG of group III (*p* = 0.002) only, showing an increase of muscle thickness with the increase of ankle plantarflexion (Figure 5). 

## 4. Discussion

For a better understanding of the potentially altered architecture of the triceps surae muscle complex, thorough evaluation using subjects with a homogenous disease entity and no confounding factors in a study are the first requirement. Furthermore, in vivo observations of the muscle behavior should be performed under the control of the joint positions at the knee and ankle, as the triceps muscles are composed of one mono-articular and two bi-articular muscles. On the basis of the findings obtained in the present study, we confirmed that the changes detected in the architectural properties of the three muscles of the triceps surae induced by systematic variations of the position at the ankle and the knee joints were variable to different degrees. Since the structural changes can be encountered in the gastrocnemius as well as in both the gastrocnemius and the soleus, it may be necessary to consider the interactions between the muscles when considering the operative procedure to correct the equinus deformity. All the above-mentioned factors indicate that the original Silfverskiöld test should be reappraised in terms of potential interactions between the muscles of the triceps surae during varying degrees of flexion/extension at the ankle and the knee joints. 

Firth et al. subdivided the triceps complex into three zones and found that for each, different types of surgery varied in terms of selectivity, stability, and range of correction [33]. In addition, procedures for the correction of equinus were noted to have different anatomical and biomechanical characteristics. For example, in the zone extending from the femoral origins of the gastrocnemius muscle to the distal extent of the gastrocnemius muscle belly or the most distal extent of the plane of separation, intramuscular lengthening of the gastrocnemius and soleus performed in the interval between the gastrocnemius and soleus fascia may lengthen the gastrocnemius selectively. They also concluded that differential lengthening of the gastrocnemius and soleus may be performed by varying the number of cuts in the fascia overlying the two muscles. We agree with the original concept of the Silfverskiöld test, in that it is helpful to differentiate isolated gastrocnemius equinus from dual gastrocnemius and soleus tightness [1,2,8]. However, we think that the choice of an appropriate surgical procedure for rectifying the equinus deformity may not be determined solely based on the original suggestions by Silfverskiöld.

In the current study, we used the Silfverskiöld test and performed ultrasonographic evaluation of the triceps muscles in normal children and hemiplegic patients. The advantage of using ultrasonography is that the changes in behavior of the fascicles in each muscle can be detected *in vivo*. Previous studies reported inconsistent findings regarding the muscle architecture in children with CP and equinus deformity [23,24,25,26,27,34]. For the gastrocnemii, Shortland et al. [26] reported that children with diplegia have a fascicle length which is similar to that of typically-developing children. However, others found a shorter fascicle length in patients with diplegia [22,25]. We think that this discrepancy came from the fact that past studies did not control for confounding factors such as skeletal deformities (planovalgus, tibial/femoral torsional deformity, and knee flexion deformity) which are commonly seen in the diplegics. In this study, we included only type II hemiplegic patients without skeletal abnormalities in order to exclude any conditions leading to distortion in muscle integrity. 

Mohagheghi et al. [24] and Chen et al. [19] suggested that the reduced fascicle length and muscle thickness in the gastrocnemius are possible causes of equinus deformity in hemiplegics. However, they measured the fascicle length in the resting position only. In the resting position, fascicle length in children with equinus deformity would be decreased due to increased ankle plantarflexion. We compared fascicle lengths between the groups under the same joint angle, and we noted diverse changes in the fascicle length of paretic side in children with hemiplegia; fascicle lengths were similar in MG, higher in LG, and lower in SOL. In individuals without neuro-musculo-skeletal disorders, the fascicle length of the lateral gastrocnemius is the longest of the triceps surae [11]. MG muscle belly is longer than that of the LG, and the differences in force-producing capabilities of the triceps surae is related to the different changes of pennation angle and fascicle length in each muscle during the contraction [35]. Our results suggest that MG, LG, and SOL are affected by spasticity to differing extents. 

**Figure 6:**
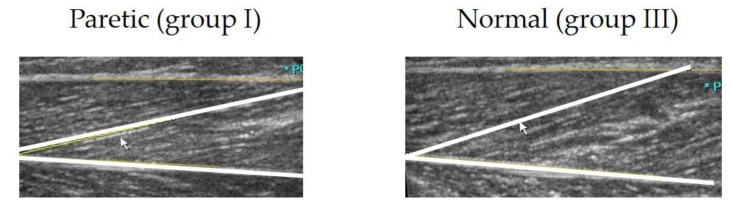
Sonographic images of the lateral gastrocnemius. Pennation angle and muscle thickness in paretic legs (16° and 8 mm, respectively) were found to be decreased, compared to those in normal legs of typically developing children (22° and 12 mm, respectively) at the same joint angle.

Malaiya et al. [23] suggested that equinus in children with hemiplegia may not be due to a decrease in fascicle length of the gastrocnemii. Barber et al. [28] demonstrated that the equinus is rather related to a lack of cross-sectional growth and decreased muscle volume of the medial gastrocnemius. Although muscle thickness measured in our study was only an estimation of muscle volume, differences between the groups indicated the presence of substantial muscle atrophy in paretic legs, consistent with previous reports [9,24,34]. In addition, pennation angles of both the medial and lateral gastrocnemii were significantly smaller in paretic legs, which is in line with the previous findings of a larger pennation angle in hypertrophied muscles and increase after gastrocnemius lengthening or botulinum toxin injection [15,27,36]. Recent studies on the muscle architecture described cerebral palsy as a short/small muscle disease, and overstretched sarcomeres have been suggested as one of the reasons for muscle weakness in children with CP [10,20,21,37,38]. Our results indicate that gastrocnemius in hemiplegics has a longer or similar fascicle length, a smaller pennation angle, and a reduced muscle thickness compared to the muscles in normal children (Figure 6).

In isometric contraction of the triceps in healthy individuals, soleus’s fascicle behavior was suggested to depend on the ankle joint angle tested but not on the knee joint angle [11,16,34]; these observations may provide evidence that architectural properties of the soleus is not influenced by the knee joint angle. On the contrary, there were observations of differential displacement of the soleus and medial gastrocnemius aponeuroses during isometric contractions [7] and difference of fascicle’s behavior between the medial gastrocnemius and soleus during walking [6]. In the current study, fascicle length of the soleus in a normal leg increased with the increase of knee flexion and fascicle length of the gastrocnemii increased with the increase of knee extension; and pennation angle of the soleus in hemiplegics increased with the increase of knee extension and pennation angle of the medial gastrocnemius increased with the increase of knee flexion. The Silfverskiöld test is not a voluntary test but an examination being performed passively by the examiner; thus, the assumption that only the gastrocnemius is influenced by passive knee flexion during the Silfverskiöld test needs to be reconsidered. 

This study has several limitations that have to be considered when interpreting the results. The data presented in the current study could be misleading due to the small number of the subjects enrolled, and generalization of our data would have been more accurate with larger cohorts. We excluded individuals older than 10 years of age, considering their rapid growth rate. In addition, those children with CP have tendencies of having more deformities developed in the coronal, sagittal, and/or transverse planes. The individuals younger than 5 years of age were also excluded because many had had botulinum toxin injection before the study and their leg muscles are too small to examine. In the present study, we enrolled children aged 5–10 years old. Growth gradually slows until reaching a steady velocity at the age of 4 to 5 and this slow and constant rate continues until puberty [39], and the fascicle angle was found to be unchanged in this age group [40]. In addition, children with equinus deformity only are more prevalent in this age group in CP. In order to examine the nature of the Silfverskiöld test, we believed that the inclusion of patients with only a sagittal plane deformity at the ankle was a priority because even subtle spasticity of the hamstrings may affect the excursion of gastrocnemius muscle. In order to exclude any confounding factors of the analysis of the integrity of the triceps surae muscles complex, we included patients with a homogenous condition of spastic hemiplegia in terms of the degrees of neurologic involvement and ambulatory status. Our findings may not be reproducible in complicated patients with skeletal deformities as seen in many of the diplegic patients. Secondly, we did not appreciate the effects of increased echo intensity which may be seen in patients with CP. Increased echo intensity may act as a bias during measurement, however, this increase of echo intensity was found to be less in patients with the mobility of GMFCS level I as in our series [41]. In addition, although we followed the standard protocol of probe positioning to decrease the measurement error [32], it should be noted that measured fascicle length and pennation angle may still be underestimated or overestimated, as observed in a study using simulated ultrasound images based on three-dimensional (3D) models of the medial gastrocnemius, derived from magnetic resonance and diffusion tensor images [42].

Further studies will be needed to assess potential tissue stiffness encountered in cerebral palsy and its influence on the alterations of the muscle architectures to examine any correlation between the findings obtained in the static examination and the dynamic changes in real walking. Also, as the conventional ultrasound images of muscle fascicles account for two-dimensional (2D) analysis of the aponeurosis displacement during tensile loading, studies on any unaccounted deformation occurring in the additional plane may need to be followed; for example, the architecture and morphology of the muscles can be visible within the 3D images that are generated by a technique using 2D B-mode ultrasound and a motion tracking system, and such method may be more feasible in extracting muscle and tendon lengths to analyze the nature of the Silfverskiöld test [43]. 

## 5. Conclusions

In the present study, we found the altered architecture of triceps surae muscles in patients with spastic hemiplegia and equinus deformity. Compared to the results of measurements in the contralateral non-paretic or normal leg, the fascicle length was not different in the medial gastrocnemius of a paretic leg, but it was longer in the lateral gastrocnemius and shorter in the soleus; the pennation angle was smaller in both medial and lateral gastrocnemii, and was not different in the soleus; and muscle thickness was found to be reduced in the three muscles of the paretic leg. Contrary to the observations in both the medial and lateral gastrocnemii, the fascicle length was increased and the pennation angle was decreased in the soleus with an increase of knee flexion. We believe that the limited utility of the Silfverskiöld test should be considered in patients with altered architecture of the triceps muscles in conditions such as cerebral palsy, as the differing muscle architectures of the triceps surae muscles complex may affect the behavior of the muscles during the Silfverskiöld test. The choice of an appropriate surgical procedure for rectifying the equinus deformity may not be determined solely based on the original suggestions by Silfverskiöld.

## Figures and Tables

**Table 1:** Details of the patients.

	ID	Sex	Age (Month)	Height (cm)	Weight (kg)	Femoral Anteversion (°)		Tibial Torsion (°)	
						Rt	Lt	Rt	Lt
SHCP	1	M	87	135	40	18	10	22	18
	2	M	60	110	19	10	16	5	14
	3	M	60	105	18	23	22	29	19
	4	M	66	112	14	16	28	15	14
	5	M	60	118	23	14	22	16	23
	6	F	20	105	14	6	16	22	16
	7	F	109	163	55	12	24	20	22
	8	F	121	155	49	17	10	14	18
	9	F	71	112	18	20	11	17	26
	10	M	70	104	15	5	11	13	11
Normal children	11	M	83	124	23	8	9	17	20
	12	M	62	114	21	20	22	21	20
	13	M	85	131	26	18	20	20	23
	14	M	85	125	34	10	12	15	18
	15	M	84	126	26	13	11	16	18
	16	F	96	116	20	19	22	19	22
	17	F	88	135	34	17	16	21	24
	18	F	73	112	20	20	19	17	20
	19	F	76	128	22	16	14	23	22
	20	M	92	116	21	8	8	15	17

ID 1–10, SHCP (spastic hemiplegic cerebral palsy); ID 11–20, normal children; M, male; F, female; Rt, right; L, left.

## Supplementary Materials

The followings are available online at https://www.mdpi.com/2077-0383/8/12/2096/s1. Figure S1: to prevent the influence of midfoot motion on the measurement of ankle dorsiflexion, the Silfverskiöld test is performed with the forefoot supinated. Figure S2: fascicle length of the medial gastrocnemius (MG), lateral gastrocnemius (LG), and soleus (SOL) muscles in each cases. A-10, A00, A15, and A30 represent 10° ankle dorsiflexion, ankle neutral, 15° plantarflexion, and 30° plantarflexion, respectively; while K00, K45, and K90 represent full knee extension, 45° knee flexion, and 90° knee flexion, respectively. Figure S3: pennation angle of the medial gastrocnemius (MG), lateral gastrocnemius (LG), and soleus (SOL) muscles in each cases. A-10, A00, A15, and A30 represent 10° ankle dorsiflexion, ankle neutral, 15° plantarflexion, and 30° plantarflexion, respectively; while K00, K45, and K90 represent full knee extension, 45° knee flexion, and 90° knee flexion, respectively. Figure S4: muscle thickness of the medial gastrocnemius (MG), lateral gastrocnemius (LG), and soleus (SOL) muscles in each case. A-10, A00, A15, and A30 represent 10° ankle dorsiflexion, ankle neutral, 15° plantarflexion, and 30° plantarflexion, respectively; while K00, K45, and K90 represent full knee extension, 45° knee flexion, and 90° knee flexion, respectively. Table S1: Lf (mm), Pa (degree) and T (mm).

## References

[1] Silfverskiöld N. (1924). Reduction of the uncrossed two-joints muscles of the leg to one-joint muscles in spastic conditions. Acta Chir. Scand..

[2] Singh D. (2013). Nils Silfverskiöld (1888-1957) and gastrocnemius contracture. Foot Ankle Surg..

[3] Perry J., Hoffer M.M., Giovan P., Antonelli D., Greenberg R. (1974). Gait analysis of the triceps surae in cerebral palsy. A preoperative and postoperative clinical and electromyographic study. J. Bone Jt. Surg. Am..

[4] Wren T.A.L., Cheatwood A.P., Rethlefsen S.A., Hara R., Perez F.J., Kay R.M. (2010). Achilles tendon length and medial gastrocnemius architecture in children with cerebral palsy and equinus gait. J. Pediatr. Orthop..

[5] Gao F., Zhao H., Gaebler-Spira D., Zhang L.Q. (2011). In vivo evaluations of morphologic changes of gastrocnemius muscle fascicles and achilles tendon in children with cerebral palsy. Am. J. Phys. Med. Rehabil..

[6] Ishikawa M., Komi P.V., Grey M.J., Lepola V., Bruggemann G.P. (2005). Muscle-tendon interaction and elastic energy usage in human walking. J. Appl. Physiol..

[7] Bojsen-Møller J., Hansen P., Aagaard P., Svantesson U., Kjaer M., Peter Magnusson S.P. (2004). Differential displacement of the human soleus and medial gastrocnemius aponeuroses during isometric plantar flexor contractions in vivo. J. Appl. Physiol..

[8] DiGiovanni C.W., Kuo R., Tejwani N., Price R., Hansen S.T., Cziernecki J., Sangeorzan B.J. (2002). Isolated gastrocnemius tightness. J. Bone Jt. Surg. Am..

[9] Lieber R.L., Steinman S., Barash I.A., Chambers H. (2004). Structural and functional changes in spastic skeletal muscle. Muscle Nerve.

[10] Multani I., Manji J., Tang M.J., Herzog W., Howard J.J., Graham H.K. (2019). Sarcopenia, Cerebral Palsy, and Botulinum Toxin Type A. J. Bone Jt. Surg. Rev..

[11] Kawakami Y., Ichinose Y., Fukunaga T. (1998). Architectural and functional features of human triceps surae muscles during contraction. J. Appl. Physiol..

[12] Wakahara T., Kanehisa H., Kawakami Y., Fukunaga T. (2009). Effects of knee joint angle on the fascicle behavior of the gastrocnemius muscle during eccentric plantar flexions. J. Electromyogr. Kinesiol..

[13] Fukunaga T., Ichinose Y., Ito M., Kawakami Y., Fukashiro S. (1997). Determination of fascicle length and pennation in a contracting human muscle in vivo. J. Appl. Physiol..

[14] Chow R.S., Medri M.K., Martin D.C., Leekam R.N., Agur A.M., McKee N.H. (2000). Sonographic studies of human soleus and gastrocnemius muscle architecture: Gender variability. Eur. J. Appl. Physiol..

[15] Kawakami Y., Abe T., Fukunaga T. (1993). Muscle-fiber pennation angles are greater in hypertrophied than in normal muscles. J. Appl. Physiol..

[16] Maganaris C.N., Baltzopoulos V., Sargeant A.J. (1998). In vivo measurements of the triceps surae complex architecture in man: Implications for muscle function. J. Physiol..

[17] Lorenzo T.M., Rocon E., Caballero I.M., Lara S.L. (2018). Medial gastrocnemius structure and gait kinetics in spastic cerebral palsy and typically developing children a cross-sectional study. Medicine.

[18] Lorenzo T.M., Rodríguez G.A., Rocon E., Caballero I.M., Lara S.L. (2017). Relationship of medial gastrocnemius relative fascicle excursion and ankle joint power and work performance during gait in typically developing children: A cross-sectional study. Medicine.

[19] Chen Y., He L., Xu K., Li J., Guan B., Tang H. (2018). Comparison of calf muscle architecture between Asian children with spastic cerebral palsy and typically developing peers. PLoS ONE.

[20] Noble J.J., Fry N.R., Lewis A.P., Keevil S.F., Gough M., Shortland A.P. (2014). Lower limb muscle volumes in bilateral spastic cerebral palsy. Brain Dev..

[21] Herskind A., Ritterband-Rosenbaum A., Willerslev-Olsen M., Lorentzen J., Hanson L., Lichtwark G., Nielsen J.B. (2016). Muscle growth is reduced in 15-month-old children with cerebral palsy. Dev. Med. Child Neurol..

[22] Kruse A., Schranz C., Tilp M., Svehlik M. (2018). Muscle and tendon morphology alterations in children and adolescents with mild forms of spastic cerebral palsy. BMC Pediatr..

[23] Malaiya R., McNee A.E., Fry N.R., Eve L.C., Gough M., Shortland A.P. (2007). The morphology of the medial gastrocnemius in typically developing children and children with spastic hemiplegic cerebral palsy. J. Electromyogr. Kinesiol..

[24] Mohagheghi A.A., Khan T., Meadows T.H., Giannikas K., Baltzopoulos V., Maganaris C.N. (2007). Differences in gastrocnemius muscle architecture between the paretic and non-paretic legs in children with hemiplegic cerebral palsy. Clin. Biomech..

[25] Mohagheghi A.A., Khan T., Meadows T.H., Giannikas K., Baltzopoulos V., Maganaris C.N. (2008). In vivo gastrocnemius muscle fascicle length in children with and without diplegic cerebral palsy. Dev. Med. Child Neurol..

[26] Shortland A.P., Harris C.A., Gough M., Robinson R.O. (2002). Architecture of the medial gastrocnemius in children with spastic diplegia. Dev. Med. Child Neurol..

[27] Shortland A.P., Fry N.R., Eve L.C., Gough M. (2004). Changes to medial gastrocnemius architecture after surgical intervention in spastic diplegia. Dev. Med. Child Neurol..

[28] Barber L., Hastings-Ison T., Baker R., Barrett R., Lichtwark G. (2011). Medial gastrocnemius muscle volume and fascicle length in children aged 2 to 5years with cerebral palsy. Dev. Med. Child Neurol..

[29] Palisano R., Rosenbaum P., Walter S., Russell D., Wood E., Galuppi B. (1997). Development and reliability of a system to classify gross motor function in children with cerebral palsy. Dev. Med. Child Neurol..

[30] Winters T.F., Gage J.R., Hicks R. (1987). Gait patterns in spastic hemiplegia in children and young adults Patterns in Spastic and Young Hemiplegia Adults. J. Bone Jt. Surg. Am..

[31] Martin D.C., Medri M.K., Chow R.S., Oxorn V., Leekam R.N., Agur A.M., McKee N.H. (2001). Comparing human skeletal muscle architectural parameters of cadavers with in vivo ultrasonographic measurements. J. Anat..

[32] Bénard M.R., Becher J.G., Harlaar J., Huijing P.A., Jaspers R.T. (2009). Anatomical information is needed in ultrasound imaging of muscle to avoid potentially substantial errors in measurement of muscle geometry. Muscle Nerve.

[33] Firth G.B., McMullan M., Chin T., Ma F., Selber P., Eizenberg N., Wolfe R., Graham H.K. (2013). Lengthening of the gastrocnemius-soleus complex: An anatomical and biomechanical study in human cadavers. J. Bone Jt. Surg. Am..

[34] Fry N.R., Gough M., McNee A.E., Shortland A.P. (2007). Changes in the volume and length of the medial gastrocnemius after surgical recession in children with spastic diplegic cerebral palsy. J. Pediatr. Orthop..

[35] Antonios T., Adds P.J. (2008). The medial and lateral bellies of gastrocnemius: A cadaveric and ultrasound investigation. Clin. Anat..

[36] Park E.S., Sim E., Rha D.W., Jung S. (2014). Architectural Changes of the Gastrocnemius Muscle after Botulinum Toxin Type an Injection in Children with Cerebral Palsy. Yonsei Med. J..

[37] Mathewson M.A., Lieber R.L. (2015). Pathophysiology of muscle contractures in cerebral palsy. Phys. Med. Rehabil. Clin. N. Am..

[38] Fortuna R., Vaz M.A., Sawatsky A., Hart D.A., Herzog W. (2015). A clinically relevant BTX-A injection protocol leads to persistent weakness, contractile material loss, and an altered mRNA expression phenotype in rabbit quadriceps muscles. J. Biomech..

[39] Morcuende J.A., Sanders J.O., Weinstein S.L., Flynn J.M. (2014). Embryology and Development of the Neuromuscular Apparatus. Lovell and Winter’s Pediatric Orthopaedics.

[40] Bénard M.R., Harlaar J., Becher J.G., Huijing P.A., Jaspers R.T. (2011). Effects of growth on geometry of gastrocnemius muscle in children: A three-dimensional ultrasound analysis. J. Anat..

[41] Pitcher C.A., Elliott C.M., Panizzolo F.A., Valentine J.P., Stannage K., Reid S.L. (2015). Ultrasound characterization of medial gastrocnemius tissue composition in children with spastic cerebral palsy. Muscle Nerve.

[42] Bolsterlee B., Gandevia S.C., Herbert R.D. (2016). Effect of Transducer Orientation on Errors in Ultrasound Image-Based Measurements of Human Medial Gastrocnemius Muscle Fascicle Length and Pennation. PLoS ONE.

[43] Fry N.R., Gough M., Shortland A.P. (2004). Three-dimensional realization of muscle morphology and architecture using ultrasound. Gait Posture.

