# Supplementary material for: Architecture of the Triceps Surae Muscles Complex in Patients with Spastic Hemiplegia: Implication for the Limited Utility of the Silfverskiöld Test
Source: J Clin Med. 2019 Dec 1;8(12):2096. doi: 10.3390/jcm8122096 (PMC6947161; doi:10.3390/jcm8122096)
Supplement: Supplementary file 1 [file jcm-08-02096-s001.pdf]

Supplementary Materials:

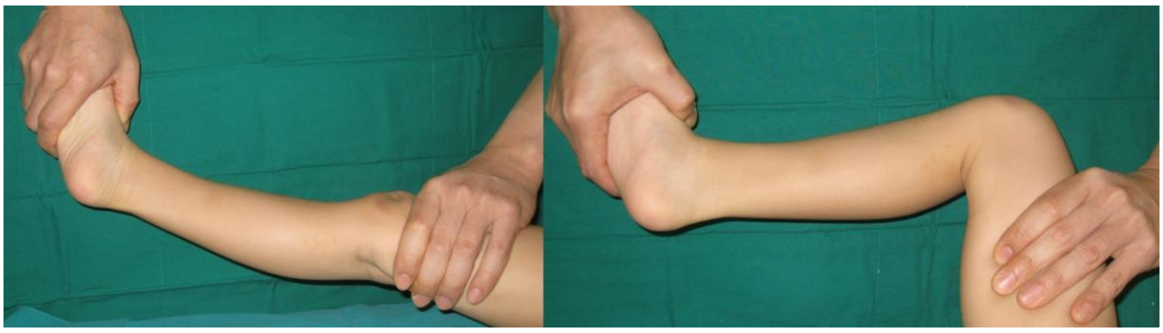

Figure S1. to prevent the influence of midfoot motion on the measurement of ankle dorsiflexion, the Silfverskiöld test is performed with the forefoot supinated.

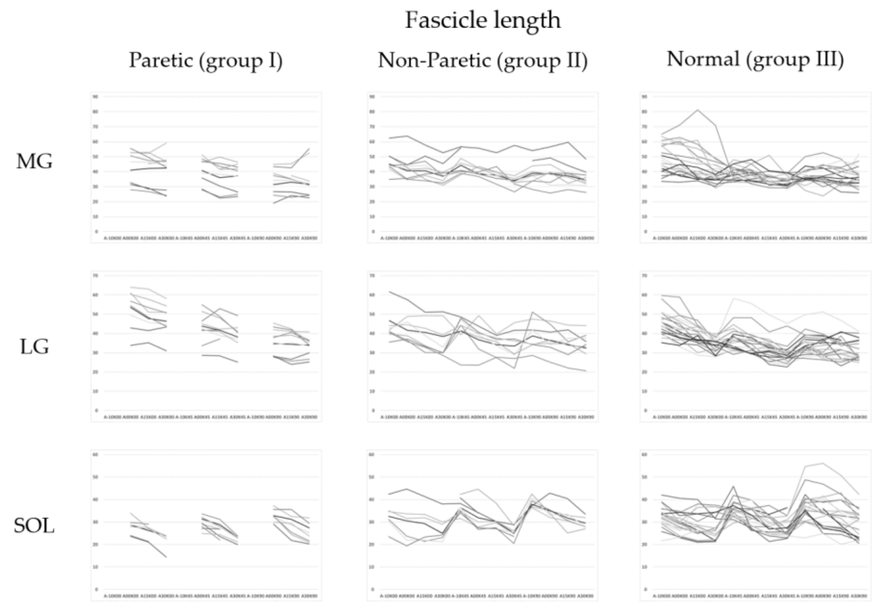

Figure S2. fascicle length of the medial gastrocnemius (MG), lateral gastrocnemius (LG), and soleus (SOL) muscles in each cases. A-10, A00, A15, and A30 represent 10˚ ankle dorsiflexion, ankle neutral, 15˚ plantarflexion, and 30˚ plantarflexion, respectively; while K00, K45, and K90 represent full knee extension, 45˚ knee flexion, and 90˚ knee flexion, respectively.

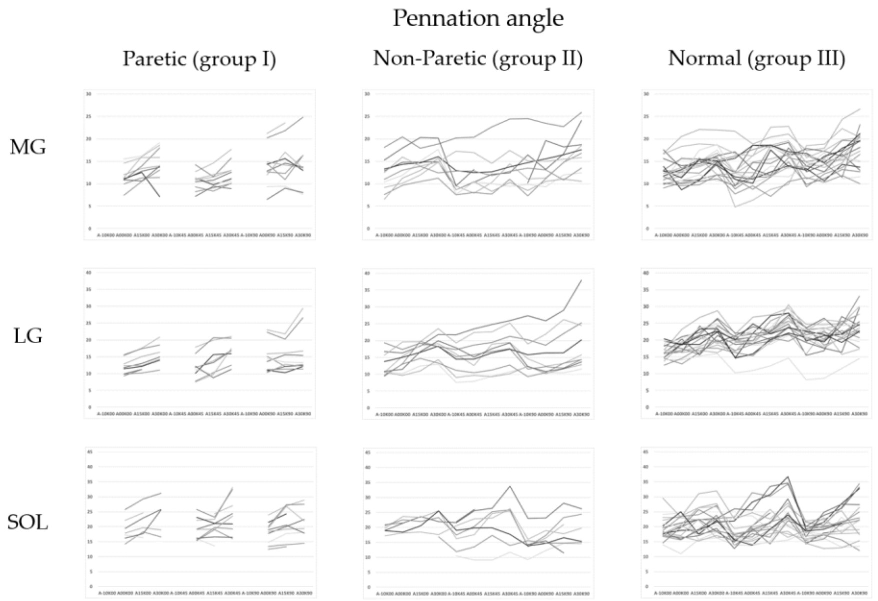

Figure S3. pennation angle of the medial gastrocnemius (MG), lateral gastrocnemius (LG), and soleus (SOL) muscles in each cases. A-10, A00, A15, and A30 represent 10˚ ankle dorsiflexion, ankle neutral, 15˚ plantarflexion, and 30˚ plantarflexion, respectively; while K00, K45, and K90 represent full knee extension, 45˚ knee flexion, and 90˚ knee flexion, respectively.

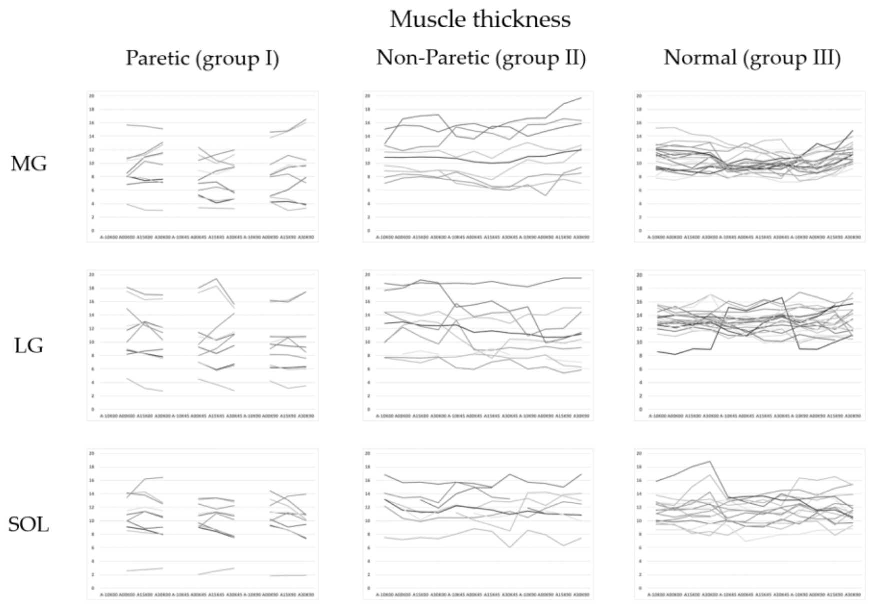

Figure S4. muscle thickness of the medial gastrocnemius (MG), lateral gastrocnemius (LG), and soleus (SOL) muscles in each case. A-10, A00, A15, and A30 represent 10° ankle dorsiflexion, ankle neutral, 15° plantarflexion, and 30° plantarflexion, respectively; while K00, K45, and K90 represent full knee extension, 45° knee flexion, and 90° knee flexion, respectively.

Table S1. Lf (mm), Pa (degree) and T (mm)

Knee angle			0°				45° flexion				90° flexion			
Ankle angle			10° DF	0° PF	15° PF	30° PF	10° DF	0° PF	15° PF	30° PF	10° DF	0° PF	15° PF	30° PF
Lf	MG	Gr I		42 ± 12.6	43.4 ± 11.3	42.6 ± 12.4		39.9 ± 10	36.4 ± 8.4	37.3 ± 8		32.9 ± 9.8	33.6 ± 6.5	31.9 ± 9.3
		Gr II	44.8 ± 8.2	41 ± 9.6	41.5 ± 9.3	38.3 ± 8.2	44.9 ± 9.4	41.7 ± 11	37.7 ± 7.2	34.8 ± 9.9	39.5 ± 7.3	38.5 ± 8.7	36.8 ± 8.9	34.4 ± 7.4
		Gr III	46 ± 8.1	45.4 ± 8.4	42.9 ± 9.4	38.5 ± 7.5	40 ± 4.5	38.4 ± 4.4	35.4 ± 3.9	33.8 ± 3.6	36.7 ± 4.5	36.5 ± 5.4	36.1 ± 5.7	35.3 ± 6.2
	LG	Gr I		48.2 ± 12	46.2 ± 9.8	44.6 ± 9.3		43.2 ± 9.2	40.3 ± 8.2	36.8 ± 8.3		34.5 ± 8.2	34.7 ± 7.2	33.7 ± 7.5
		Gr II	46.7 ± 10.3	43.1 ± 6.4	41.6 ± 9.8	38.8 ± 9.6	42.8 ± 7.1	38.4 ± 7.3	35.9 ± 5.3	34.8 ± 7.2	36.6 ± 7	35.6 ± 7.2	34 ± 9.1	32.5 ± 8.4
		Gr III	44.7 ± 5.4	41.1 ± 4.7	37.9 ± 4.2	33.9 ± 3.5	37.9 ± 6.4	35.1 ± 6.4	32.2 ± 5.9	30.3 ± 6.2	34.8 ± 5	33.6 ± 5.2	33 ± 5.4	31.1 ± 4.5
	SOL	Gr I		29.3 ± 4.8	27.9 ± 7	23.6 ± 7		31.2 ± 3.3	28.3 ± 3.7	23.6 ± 3		32.6 ± 3.1	30.6 ± 7.2	27.2 ± 5.6
		Gr II	32.6 ± 7	30.2 ± 9	29.1 ± 8.1	24.7 ± 13.4	35.3 ± 4.5	31.2 ± 5.3	30 ± 4.3	25.8 ± 2.7	36.8 ± 3.2	34.3 ± 5	31.4 ± 5.3	29.5 ± 2.9
		Gr III	34.5 ± 6.6	30.6 ± 5.9	29.2 ± 5.7	27.8 ± 5.4	35.9 ± 4	32.3 ± 3.7	29.2 ± 4.2	27.5 ± 3.8	37 ± 7.4	35.3 ± 8.2	32.7 ± 7.9	29.3 ± 6.7
Pa	MG	Gr I		11.5 ± 2.1	13.1 ± 2.7	13.8 ± 2.7		10.7 ± 2.1	11.2 ± 2.7	12.8 ± 2.5		13.9 ± 5.2	14.4 ± 4.1	14.2 ± 4.5
		Gr II	12.9 ± 3.7	14.5 ± 3.3	14.4 ± 2.9	15.6 ± 2.9	12.4 ± 4	12.4 ± 3.6	12.3 ± 4.2	14 ± 4.2	14.1 ± 4.7	15.4 ± 4.1	16 ± 3.7	18.1 ± 4.6
		Gr III	12.4 ± 2.5	12.9 ± 2.8	14.1 ± 2.7	16.1 ± 3.6	12.4 ± 3.5	13.4 ± 3.1	15.3 ± 3.7	16.3 ± 3.1	14.3 ± 2.8	14.9 ± 3	16.4 ± 3.4	18 ± 4
	LG	Gr I		12.4 ± 2.4	13.9 ± 2.5	15.4 ± 3.3		11.3 ± 4.1	13.9 ± 4.8	16.4 ± 4.6		14.8 ± 5.4	15.9 ± 3.6	16.1 ± 4.8
		Gr II	13.7 ± 3.2	14.5 ± 3.2	16.3 ± 3.3	17.8 ± 4	15.3 ± 3.9	15.4 ± 4.5	16.2 ± 5.8	17.7 ± 5.6	15.9 ± 4.8	15.8 ± 5.4	16.9 ± 6.1	19.7 ± 8.3
		Gr III	16.7 ± 2.2	17.9 ± 2.4	19.9 ± 2.6	22 ± 3	19.2 ± 2.7	20.9 ± 3.6	22.7 ± 3.4	25.3 ± 4.1	20.8 ± 4	20.8 ± 4	22.3 ± 3.6	24.3 ± 4.4
	SOL	Gr I		18.7 ± 4.6	21.7 ± 6.2	23.8 ± 5.6		19.7 ± 4.5	21 ± 3.9	24.5 ± 6.4		18.1 ± 4.1	19.2 ± 5.9	20 ± 5.1
		Gr II	16.8 ± 5.4	17.3 ± 5.1	18 ± 7	17.9 ± 7.2	19.3 ± 6.3	20.3 ± 7.5	20.3 ± 6.6	23.1 ± 9	16.1 ± 4.6	18.4 ± 6.2	17.5 ± 5.7	19.8 ± 5.2
		Gr III	18.6 ± 3.3	19.3 ± 4	21.3 ± 4.6	22.5 ± 5.9	17.5 ± 3.6	19 ± 4.9	21.5 ± 4.5	24.3 ± 6.4	17.5 ± 2.7	18.6 ± 3.6	20.3 ± 5.8	21.6 ± 6.7
T	MG	Gr I		10.3 ± 3.9	10.9 ± 3	11.5 ± 3.1		8.8 ± 3.3	8.6 ± 3.4	9.4 ± 3.3		8.5 ± 4.4	9.6 ± 3	9.6 ± 3.4
		Gr II	11.1 ± 3	11 ± 3.4	11 ± 3.6	11 ± 3.5	11.1 ± 3.2	10.2 ± 3.8	10.1 ± 3.8	10.1 ± 3.8	11.4 ± 3.7	11.2 ± 3.9	11.6 ± 4	11.9 ± 4.1

	Gr III	10.7 ± 1.8	10.7 ± 1.9	10.7 ± 1.8	10.8 ± 1.7	9.7 ± 1.2	9.9 ± 1.3	10 ± 1.4	9.9 ± 1.4	9.8 ± 1.3	10.2 ± 2.1	10.8 ± 2	11.7 ± 1.9
	Gr I		12.5 ± 4.7	12.9 ± 3.3	12.1 ± 2.8		10.9 ± 4.7	10.3 ± 3.7	11.3 ± 2.7		10 ± 4.2	10.7 ± 3.1	10.7 ± 3.5
LG	Gr II	12.9 ± 4.3	12.9 ± 4.5	12.6 ± 4.2	12.5 ± 4.3	12.5 ± 3.9	11.4 ± 3.9	11.6 ± 4	11.3 ± 3.5	10.9 ± 3.7	10.8 ± 3.6	10.8 ± 4.4	11.3 ± 4.9
	Gr III	13.1 ± 1.6	12.9 ± 1.5	12.8 ± 1.3	12.9 ± 1.6	12.9 ± 1.5	12.7 ± 1.5	12.9 ± 1.8	13.2 ± 1.7	12.8 ± 2	12.8 ± 2	13 ± 1.9	13.4 ± 2
	Gr I		11.5 ± 2.6	12 ± 2.8	11.4 ± 2.9		11.1 ± 2	11.3 ± 2.4	10.8 ± 2.9		11.4 ± 2.1	11.3 ± 1.9	10.9 ± 1.9
SOL	Gr II	13.1 ± 3.3	11.7 ± 2.3	11.4 ± 2.4	11.3 ± 2.3	12.2 ± 2.9	11.9 ± 2.6	11.7 ± 2.6	11.2 ± 3.3	11.4 ± 1.9	11.1 ± 2.1	10.9 ± 2.4	10.8 ± 2.8
	Gr III	11.8 ± 2.1	11.8 ± 2.4	12 ± 2.7	11.9 ± 3.1	11.6 ± 2	11.4 ± 2.1	11.3 ± 2.1	11.5 ± 2.2	11.9 ± 2.2	12.1 ± 2.1	11.7 ± 3	11.4 ± 2.7

Gr: group; MG: medial gastrocnemius; LG: lateral gastrocnemius; SOL: soleus; Lf: fascicle length; Pa: pennation angle; T: muscle thickness; DF: dorsiflexion; PF: plantarflexion

Values are mean ± standard deviation.
